# Quantitative description of the relationship between the enhancement of distraction-suppression and brain local state alteration after transcranial direct current stimulation

**DOI:** 10.3389/fnins.2022.984893

**Published:** 2022-09-06

**Authors:** Di Zhang, Jiaojiao Liu, Li Fan, Qiang Liu

**Affiliations:** ^1^Research Center of Brain and Cognitive Neuroscience, Liaoning Normal University, Dalian, China; ^2^Institute of Brain and Psychological Sciences, Sichuan Normal University, Chengdu, China

**Keywords:** resting-state fMRI, dorsal lateral prefrontal cortex, local brain activity, distraction suppression, tDCS

## Abstract

Anodal transcranial direct current stimulation (tDCS) over the left dorsal lateral prefrontal cortex (lDLPFC) can improve distraction suppression ability, possibly by distantly regulating the connection properties of several large-scale brain networks and local brain state changes. However, little is known about the local state alteration that tDCS can induce in distant but task-related regions and the relationship between performance enhancement and local state alteration in potentially related regions, resulting in inefficient and uncertain tDCS regulation. We aimed to examine the alteration of brain local state before and after tDCS and its relationship with performance enhancement. With the within-subject design, the participants received anodal (1.5 mA) and sham tDCS at F3 (lDLPFC) for 20 min. The visual search task and resting-state functional magnetic resonance imaging (rsfMRI) were performed before and after stimulation. Anodal tDCS significantly enhanced distraction suppression. The amplitude of low-frequency fluctuation (ALFF) in the left parietal region significantly decreased, the decrement significantly positively correlated with performance enhancement after anodal tDCS. As well, the regional homogeneity (ReHo) in the left precuneus significantly increased, and the increasement significantly positively correlated with performance enhancement. Anodal tDCS over the lDLPFC can distantly modulate the local state of the brain and improve the distraction suppression ability. These two aspects are closely related and provide a direct and efficient approach to enhancing performance.

## Introduction

In a complex environment, the brain screens and processes all information. Efficient processing often presupposes the brain’s ability to suppress irrelevant information effectively. Therefore, researchers are interested in how to achieve or maintain a high-level distraction suppression. According to the cognitive control theory, top-down activated attention modulation is the main processing mode for focusing on the target and actively ignoring distractions. As the core region of higher cognitive processing, the prefrontal cortex (PFC) can regulate activities in other part of the brain by sending and receiving projections from regions involved in sensory or motor processing. Therefore, it plays a crucial role as a regulator of irrelevant information (Miller and Cohen, 2001; Gazzaley and Nobre, 2012).

As an effective non-invasive intervention, tDCS can not only significantly improve the behavioral performance by shortening the inhibition response time (Yu et al., 2015) or enhancing the distraction suppression ability (Cosman et al., 2015), but also alter the brain activity state during the extraction stage of working memory when a negative distraction occurs (Wörsching et al., 2018). Meanwhile, tDCS can modulate the activity strength of the stimulus-target area (Priori et al., 1998; Nitsche and Paulus, 2000; Merzagora et al., 2010) and the correlation between the activity in the stimulated region and the activity in the distal parts. On account of this intervention model, cognitive processing associated with regions affected can be regulated by applying tDCS on the core parts (e.g., the PFC) (Plewnia et al., 2015; Ironside et al., 2016; Sandrini et al., 2020). Furthermore, investigating the tDCS intervention on crucial brain regions, which may be in charge with high-level cognitive processing, could be significative to perform the tDCS more effectively and attains a better enhancement of behavioral performance (e.g., distraction suppression).

Previous studies suggested that anodal tDCS applied on the DLPFC could significantly modulate the connection pattern of the large-scale brain network compared with the sham stage. Anodal tDCS significantly increased the coactivation of the default mode network (DMN) and frontoparietal network (Keeser et al., 2011). Another study showed that tDCS (conducted on lDLPFC) could also induce a pairwise-level change in connectivity between the lDLPFC and the junction of the bilateral superior parietal lobule and inferior parietal lobule, which suggested that tDCS can also alter the connectivity between two specific brain regions (Mondino et al., 2020). Furthermore, recent studies suggested that tDCS over the DLPFC could help modify the regional and global brain network dynamics by increasing network flexibility (Zhou et al., 2020) and the characteristics of the lDLPFC as a subregion also could be modulated by tDCS at different network scales (as a subregion of the frontoparietal control network, i.e., FPCN, or as a part out of FPCN) (Kim et al., 2021). In addition, the local blood oxygenation level-dependent (BOLD) signal activation in regions away from the stimulated area may show a significant change during the stop-signal task (Sandrini et al., 2020), which may indicate that with the connection property or connectivity pattern changes induced by tDCS, the local state of the distal areas could also be affected.

However, despite the numerous studies have suggested that tDCS can modulate the connection properties of large-scale brain networks by a cross-region intervention, to what extent the tDCS can modulate the local states of the distal regions from target is fairly unknown. As a complex processing system, regions of the brain are densely connected but relatively independent to a certain extent (e.g., showing characteristics of functional specificity or modularity) (Betzel et al., 2016). In addition to the synergistic relationship between the different brain regions, each local area is relatively independent. The DLPFC can be deeply involved in diversified cognitive processing; thus, behavioral performance (e.g., distraction suppression) may not be regulated solely by the stimulated DLPFC but rather by interactions between DLPFC and other distant areas. Therefore, it is also important to examine local state changes in the brain, particularly in the regions related to the cognitive task we want to investigate further. As far as we know, although many studies have been conducted on the effects of tDCS on distraction suppression ability (Tremblay et al., 2014), only a few studies have examined the local state changes in those areas, which distal from the stimulated region and may be related to overcoming distractions.

In this study, we aimed to examine the local state changes in the regions distal from the stimulated area before and after tDCS. We selected amplitude of low-frequency fluctuation (ALFF) and regional homogeneity (ReHo) as indicators, both of which can be considered as non-invasive brain metabolism proxy (Aiello et al., 2015; Salvia et al., 2019). ALFF can directly measure the spontaneous activity of a single brain region, which indicates local metabolic changes associated with the BOLD signal (Zang et al., 2007; Cabral-Calderin et al., 2016). ReHo can reflect the degree of activity consistency in a specific brain area. High ReHo may indicate great synchronized oscillation of neurons in the local brain region, which is not only the manifestation of the relative independence of each brain region but also the direct evidence that each independent brain region acts spontaneously (Zang et al., 2004; Kong et al., 2017). Furthermore, we detected the relationship between the performance change and local state alteration in areas related to overcoming irrelevant information, which may help modulate distraction suppression performance directly. To address these questions, the same group of participants received both anodal and sham stimulation in a random order at the lDLPFC (over the F3 point). The visual search task and rsfMRI scanning were performed both before and after stimulation. Based on previous studies, we believe that tDCS can alter the local states of brain regions in charge with distraction suppression by performing modulation on the lDLPFC. The local state alteration of the regions in the dorsal attention and visual networks, which are mainly responsible for top-down attention modulation, may be closely correlated with the change in distraction suppression ability.

## Materials and methods

### Participants

Thirty-four healthy college students (16 women; mean ± SD age, 21.8 ± 2.4 years) participated in a total of 136 fMRI sessions (4 sessions per participant). All the participants had normal or corrected normal visual acuity and had no visual impairments such as achromatopsia and hypochromatopsia. They were informed of the experimental procedure before signing the informed consent form and kept blinded to the treatment conditions. All procedures involving human participation had been carried out in accordance with the Declaration of Helsinki and were approved by the ethics committee of the Liao Ning Normal University Research Center of Brain and Cognitive Neuroscience. The participants received monetary compensation for participating in the study.

### Transcranial direct current stimulation

The tDCS device used in the experiment was ActivaDose II (ActivaTek Inc., CA, United States). Before stimulation, two 5 × 5cm^2^ spongy pads were soaked in normal saline solution and wrapped in rubber leather with carbon electrodes of the same size. In the experiment, the participants were anodized at the F3 position (determined with an international 10–20 electroencephalographic localization system), and the cathode was placed on the contralateral cheek (Hsu et al., 2014). The participants were told that any discomfort such as unilateral instantaneous phosphene should be reported immediately. The intensity of the constant current was 1.5 mA (0.06 mA/cm^2^) for 20 min with a 30-s ramping period at the beginning and end of the stimulation (Antal et al., 2017). In the sham stage, the current intensity would increase to 1.5 mA steadily over the first 30 s, and then the electrical stimulator would be turned off without the participants’ awareness for 20 min. During both stimulation stages (anodal and sham), participants kept relax to avoid interference excitements.

### Experimental procedure

The experiment was designed as a single-blind crossover procedure. All 34 participants completed both stages (anodal and sham), which were identical except for the different electrical stimulations applied in random order.

At each stage, the participants were arranged to complete a set of pre- and post- tDCS experiment (Kim et al., 2021). At first, the participants underwent 8-min rsfMRI and 5-min T1 structural imaging, followed by the first visual attention search task. Thereafter, a 20-min tDCS (anodal or sham) was performed on F3. Then, the participants immediately returned to complete the second MRI session, after which they were required to complete the visual search task again. The visual attention search task and tDCS procedure were performed in a silent room beside the MRI room. Seven days later, they returned to complete the experiment at the other stage. The scanning procedure was performed only after safety confirmation. At the beginning of the scanning, the participants were asked to keep their heads still and their brains free of deliberate thought during the resting period. To alleviate fatigue during the scanning, they were asked to focus their eyes on a cross (2°) at the center of the screen in front of them and blink normally but try to avoid closing their eyes for a long time and falling asleep.

### Visual attention search task

**FIGURE 1:**
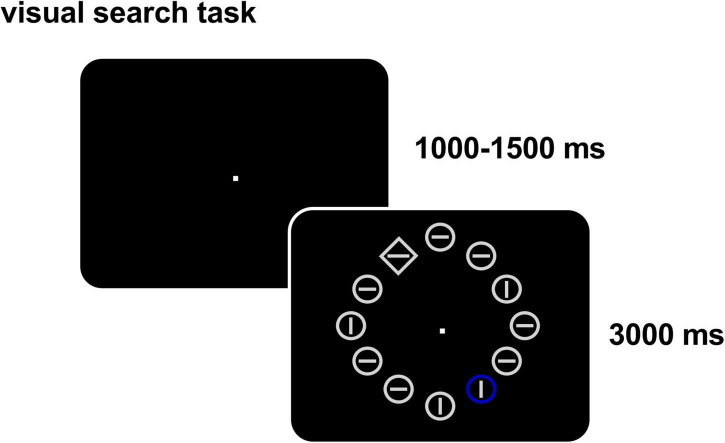
Visual search task (with a distractor). In each trial, twelve items appear and participants need to determine accurately and quickly whether the straight line in the target (with different shape) is horizontal or vertical and ignore the distractor item (different color, present in 50% of the trials).

We used the classic attentional capture paradigm (Theeuwes, 1992; Cosman et al., 2015) and the task flow is depicted in Figure 1. A bright gray circular fixation point of 1,000–1,500 ms appeared randomly at the center of the screen with a black background. Twelve stimulus items then appeared evenly (1°) on the virtual circumference (7° radius) of the screen, each consisting of a bright gray outer circumference (shape: diamond, 1.5° × 1.5°/circle, 0.8°radius) and a bright gray inner line (direction: horizontal/vertical, 1.4°). One item, which was the target item, had a different shape from the other 11 items (circus out of diamond or diamond out of circle). In distractor condition, a non-target item was colored randomly with red, yellow, blue, or green. The participants had to keep staring at the center of the screen throughout the experiment and always determine accurately and quickly whether the straight line in the target was horizontal or vertical by pressing “1” for horizontal or “2” for vertical within 3,000 ms. The experiment was divided into two blocks, each block containing 72 trials, among which those with a colored singleton distractor accounted for 50%. Each block is around 6 min, and the two blocks were performed with a total of 144 trials over a total of 12 min.

### Behavioral task analysis

The behavior data were used to calculate the accuracy rate (ACC), mean response time (RT), and distractor effect (ΔRT). The RT were calculated by averaging the response time over all trials with correct response regardless conditions in one session (two blocks). In addition, RT_with distractor_ and RT_without distractor_ were also calculated for condition “with distractor” and condition “without distractor,” respectively (e.g., RT_with distractor_ were calculated by averaging the response time over all “with- distractor trials” responded correctly), thus, ΔRT = RT_with distractor_-RT_without distractor_. To measure the response time more accurately, the error trials were excluded before calculating the mean RT and ΔRT (the percentage of trials included in each condition see Table 1). To detect whether anodal tDCS made difference on the distraction suppression ability, we also calculated the ΔΔRT in both stages (ΔΔRT = ΔRT_after stimulation_–ΔRT_before stimulation_) and performed paired-*T* test between ΔΔRT_anodal_ and ΔΔRT_sham_.

**TABLE 1:** The percentage of trials included in each condition.

		Dis		Nodis	
		Mean (%)	SD (%)	Mean (%)	SD (%)
Anodal	Before	93.97	4.41	96.73	3.03
	After	95.29	3.62	97.35	2.71
Sham	Before	94.76	4.34	96.68	3.75
	After	95.61	3.18	97.24	2.39

Dis, condition with distractor; nodis, condition without distractor; anodal, anodal stage; sham, sham stage; before, before tDCS; after, after tDCS.

### Functional magnetic resonance imaging analysis

#### Functional magnetic resonance imaging acquisition and preprocessing

Structural MRI and fMRI were performed using a GE Discovery MR750 3.0-T magnetic resonance scanner (GE Company, United States). The gradient-echo planar imaging (EPI) sequence was used to collect 240 vol of the resting state at a bottom-up interval sequence order, and the parameters were as follows: field of view (FOV), 19.2 cm; repetition time, 2,000 ms; echo time, 29 ms; flip angle, 90°; thickness, 3 mm; 43 slices and no gap; scanning matrix, 64 × 64; voxel size, 3 × 3 × 3 mm; and scanning time, 8 min. Sagittal high-precision structural imaging was also performed with a three-dimensional spoiled gradient sequence, FOV of 25.6 cm, inversion time of 450 ms, flip angle of 8°, slice thickness of 1 mm, and matrix of 256 × 256.

The rsfMRI and anatomical data were preprocessed using the DPABI toolkit^1^ with the following steps (Yan et al., 2016): After eliminating the first five volumes, all the participants’ images were slice-timing corrected and head motion corrected {subjects with motion [Mean FD Jenkinson et al. (2002)]} greater than 0.2 would be excluded (Chen et al., 2018), but no one did. Mean FD_before anodal_ = 0.051 ± 0.024, Mean FD_after anodal_ = 0.050 ± 0.028, Mean FD_before sham_ = 0.043 ± 0.018, Mean FD_after sham_ = 0.048 ± 0.028). Then nuisance signals were regressed out, including white matter signal (WM), cerebrospinal fluid signal (CSF), linear trend (for drifts in the blood oxygen level dependent signal) and signals associated with the Friston 24 head-motion parameters (i.e., 6 head motion parameters, 6 head motion parameters one time point before, and the 12 corresponding squared items) (Friston et al., 1996). As performing global signal regression is still a controversial practice in the rfMRI field, we kept the global signal for taking advantage of the whole brain information. Thereafter, the derived images were coregistered with a high-resolution T1 image individually. All the participants’ T1 images were combined to generate a T1 template using the DARTEL algorithm and normalized to the Montreal Neurological Institute (MNI) template with voxel size 3 × 3 × 3 mm^3^. Finally, the normalized images were temporal bandpass filtered (0.01-0.1Hz) to eliminate signal interference except for ALFF analyses (Buzsáki and Draguhn, 2004; Satterthwaite et al., 2013; Salvia et al., 2019), and smoothed with a 6-mm FWHM Gaussian kernel after ReHo were calculated.

#### Amplitude of low-frequency fluctuation and regional homogeneity calculation

After the calculation of ALFF and ReHo, all results were subjected to Z-standardization (Yan et al., 2013). Then, the difference map was calculated by subtracting the result before tDCS from the result after tDCS (e.g., ΔALFF = szALFF_after stimulation_-szALFF_before stimulation_, with sz indicating the ALFF map was smoothed and Z-standardization was performed; for convenience, the “sz” below was omitted).

#### Region-of-interest construction

The Dosenbach’s 160ROI template coordinates were used as the centers of the spheres with a 5-mm radius to construct spherical ROIs (Dosenbach et al., 2010). Because this study didn’t intend to discuss the state of cerebellum, we only kept the ROIs of the 142 cerebral parts for subsequent analysis (for ROIs’ information, see Supplementary file).

### Correlation analysis between ROI signals and behavioral performance

We extracted the difference signals of two resting state indicators (i.e., ΔALFF and ΔReHo) from the 142 spherical ROIs. A correlation analysis was performed between ΔΔRT and the difference signal of three indicators in 142 ROIs, respectively. For the p-values of each indicator across the ROIs, false discovery rate (FDR) correction was performed and the *FDR* < 0.05.

## Results

### Behavioral results

**FIGURE 2:**
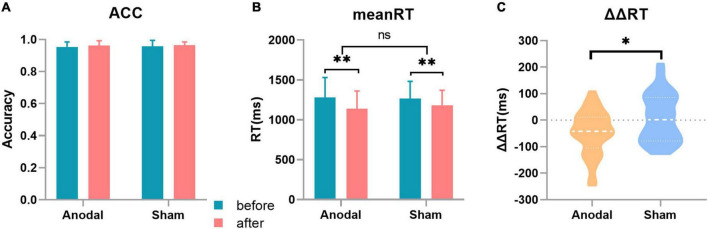
Behavior results. **(A)** Accuracy (ACC) in the two stages, with no significant changes in the different sessions or stages. **(B)** Mean response time (RT) in the two stages. **(C)** Change in the distractor effect in the two stages [distractor effect (ΔRT) = RT_with distraction_-RT_without distraction_]. Error bar: The standard deviation estimation of the sample. *p < 0.05; ^**^p < 0.01.

The results implicated that compared with the tDCS in the sham stage, the anodal tDCS on the lDLPFC significantly improved the participants’ abilities to inhibit distractors. Both stimulations (anodal or sham) can significantly shorten the response time and improved the accuracy, as depicted in Figure 2.

The mean RT, ΔRT, and ACC were tested before and after tDCS in the two stages. The results were as follows: compared with that in the sham stage, the change in the distractor effect (ΔΔRT) was significantly decreased after anodal stimulation (ΔΔRT_anodal_ = –46.64 ± 83.36 ms, ΔΔRT_sham_ = 7.42 ± 90.36 ms, *t*_33_ = 2.573, *p* = 0.015). For the mean RT, the 2 (tDCS: anodal/sham) × 2 (timepoint: before/after) analysis of variance depicted a significant main effect of tDCS (RT_before anodal_ = 1280.98 ± 249.82ms, RT_after anodal_ = 1140.16 ± 220.15ms, RT_before sham_ = 1268.24 ± 214.56ms, RT_after sham_ = 1184.18 ± 186.19ms, *F*_(1,132)_ = 8.975, *p* = 0.003) and showed no significant interaction. For the ACC, the 2 (tDCS: anodal/sham) × 2 (timepoint: before/after) analysis of variance depicted a significant main effect of tDCS (ACC_before anodal_ = 0.954 ± 0.031, ACC_after anodal_ = 0.963 ± 0.029, ACC_before sham_ = 0.957 ± 0.037, ACC_after sham_ = 0.964 ± 0.020, *F*_(1,132)_ = 4.718, *p* = 0.037) and showed no significant interaction.

### Behavior with the amplitude of low-frequency fluctuation results

**FIGURE 3:**
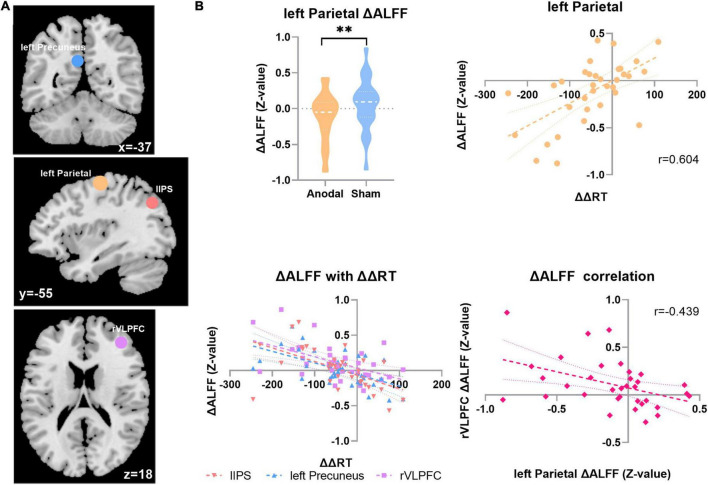
ALFF with the distractor effect results. **(A)** The ΔALFF in four ROI correlated with the ΔΔRT in two sessions in the anodal stage. Top, coronal; middle, sagittal; and bottom, axial. Blue, left precuneus; yellow, left parietal; tangerine, lIPS; violet, rVLPFC. **(B)** Top left, the ΔALFF result in the left parietal region in the two stages; top right, correlation result between ΔΔRT and ΔALFF in the left parietal region in the anodal stage; bottom left, ΔΔRT correlation result with the ΔALFF in the three regions; bottom right, ΔALFF correlation result between the rVLPFC and left parietal region. ^**^p < 0.01.

The results presented in Figure 3 show that after anodal tDCS, the change in ALFF (ΔALFF = ALFF_after stimulation_-ALFF_before stimulation_) in the left parietal region and ΔΔRT significantly positively correlated (*r*_anodal_ = 0.604; *p* = 0.0002; FDR corrected, *q* < 0.05). No such result was obtained in the sham stage. Meanwhile, as depicted upper left panel of Figure 3B, compared with the sham session, the ALFF of the left parietal region decreased significantly after anodal stimulation (ΔALFF_anodal_ = –0.118 ± 0.331, ΔALFF _sham_ = 0.064 ± 0.333, *t*_33_ = 2.704, *p* = 0.011). The ΔALFF of three other regions also showed a significant negative correlation with the change in the distractor effect, respectively, as follows: left precuneus (*r*_anodal_ = –0.571; *p* = 0.004; FDR corrected, *FDR* < 0.05), lIPS (*r*_anodal_ = –0.557; *p* = 0.006; FDR corrected, *FDR* < 0.05), and right ventrolateral PFC (rVLPFC; *r*_anodal_ = –0.513; *p* = 0.002; FDR corrected, *FDR* < 0.05), but no significant difference of ALFF changes were found in these three regions between two tDCS stages (namely, anodal tDCS stage and sham stage). Furthermore, we analyzed the relationship of spontaneous activity between the rVLPFC and the left parietal region (Figure 3). The results showed a significant negative correlation between the alterations of the ALFF in the two regions after anodal tDCS (*r*_anodal_ = –0.439, *p* = 0.0095).

### Behavior with the regional homogeneity results

**FIGURE 4:**
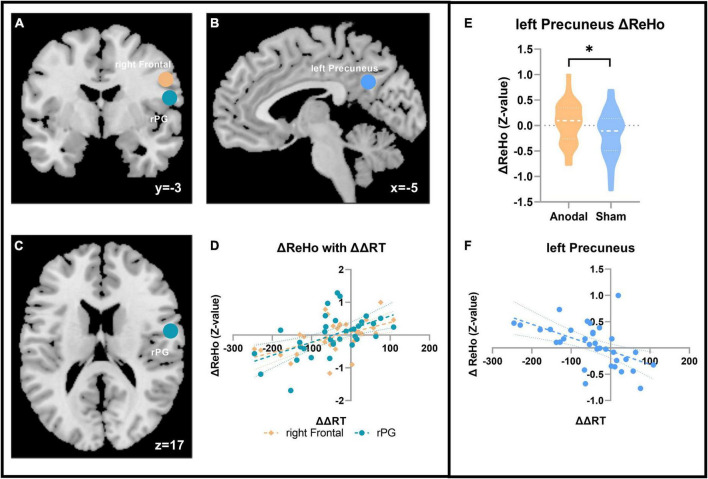
ReHo with the distractor effect results. **(A–C)** The ΔReHo in the three ROI correlated with the ΔΔRT in the two sessions in the anodal stage. **(A)** Coronal; **(B)** sagittal; and **(C)** axial. Blue, left precuneus; coffee color, right frontal; turquoise, rPG. **(D)**ΔΔRT correlation result with the ΔReHo in the two regions. **(E)** ΔReHo result in the left precuneus. **(F)** Correlation result with ΔΔRT in the left precuneus. *p < 0.05.

The results presented in Figure 4 showed that after anodal tDCS, the change in ReHo (ΔReHo = ReHo_after stimulation_-ReHo_before stimulation_) in the left precuneus was significantly negatively correlated with ΔΔRT (*r*_anodal_ = –0.536; *p* = 0.001; FDR corrected, *FDR* < 0.05). Meanwhile, compared with that in the sham stage, the ReHo after anodal tDCS increased significantly in the left precuneus (ΔReHo_anodal_ = 0.059 ± 0.397, ΔReHo_sham_ = –0.173 ± 0.455, *t*_33_ = 2.130, *p* = 0.041). In addition, the ΔReHo of two other regions also showed a significant negative correlation with the change in the distractor effect, respectively, after anodal tDCS as follows: right frontal (*r*_anodal_ = 0.523; *p* = 0.002; FDR corrected, *FDR* < 0.05) and right postcentral gyrus (rPG; *r*_anodal_ = 0.541; *p* = 0.001; FDR corrected, *FDR* < 0.05), but no significant ReHo changes were found in these two regions between two tDCS sessions.

## Discussion

In this study, tDCS was applied on the lDLPFC to examine the relationship between the changes in brain local activity state and distraction suppression ability. Combined with the basic resting state indicators (ALFF and ReHo), the results implicated that the local activity state of several brain areas changed significantly after anodal tDCS was applied on the lDLPFC. Meanwhile, distraction suppression ability was significantly enhanced. Further analysis revealed that the relationship between the distraction suppression ability and the activity state in the local areas can be quantified by analyzing the correlation between behavioral performance and resting state markers, which may provide guidance in modulating the ability to overcome irrelevant information directly.

### Changes in spontaneous activity state in the local areas and distraction suppression ability

The results of the ALFF suggested that after anodal stimulation, the alteration of spontaneous activity in the left parietal region positively correlated with the change of ΔRT, which indicates that the greater the decrease in spontaneous activity in the left parietal region is associated with the better the performance in overcoming irrelevant information (the smaller ΔRT means the better the distraction suppression performance), and this result is supported by the review from Bisley and Goldberg (2010). Evidence from primate animal studies depicted that the activity of parietal neurons is closely associated with appearance of task irrelevant items (Bisley and Goldberg, 2010). Compared with that in the sham stage, the ALFF value in the left parietal decreased significantly, showing strong coherence with the correlation results. The left parietal region lies in Brodmann area (BA) 4, which is considered a primary motor cortex (M1) and belongs to the somatomotor network (SMN), which facilitates motion processing and deals with primary sensory perception (Sanes and Donoghue, 2000; Thomas Yeo et al., 2011; Polanía et al., 2012). Prior studies have suggested that modulating M1 may regulate the elements belonging to the functional motor circuit (Polanía et al., 2012). In our study, the salient distractor with outstanding color can capture visual attention in involuntary way, which might impede the processing of distraction suppression. We believe that after anodal tDCS, this region is at a relatively low level of activation, which might be at a stage of low sensitivity to detective the salient visual item and harder to be disturbed by the distractor. This could be beneficial to overcoming interference information.

The change in ALFF in the rVLPFC, as well as in the lIPS and left precuneus region, negatively correlated with the change in the distractor effect. The rVLPFC is involved in various cognitive processes, especially attentional orientation and motor inhibition (Levy and Wagner, 2011), which may partly play a role in top-down cognitive processing. Sallard et al. (2018) found that rVLPFC exhibited more actively during the NoGo trials rather than Go trials, which might suggest that rVLPFC is in charge of inhibition and is consistent with our results. Participants in visual search task must inhibit the processing about the salient distractor, in order to search the target as quickly and accurately as possible. When rVLPFC is at a state of high-level spontaneous activity, the enhancement of distractor suppression is more significant (as ΔRT decreased more). Furthermore, the correlation result between the changes of two regions’ spontaneous activity (left parietal and rVLPFC) is also showing strong coherence with what we have discussed above. Previous studies have shown that IPS is closely related to higher cognitive activities and can be defined as attentional focalization on task-relevant information, which may lay at the lowest level of control (Majerus et al., 2016). In addition, studies also suggested that frontal lobe regions such as prefrontal cortex may transmit attentional signals and act on the IPS to initiate attention (Bressler et al., 2008). Although, in our study, the spontaneous activity in the lIPS did not change significantly after anodal tDCS, the correlation result depicted a trend that larger increasement in spontaneous activity is related to the better performance of distraction suppression. This result is consistent with previous studies and indicates that IPS may be involved in higher cognitive control processing to some extent. We deem that distraction suppression is accompanied by top-down cognitive regulation and is therefore influenced by the local states of multiple brain regions. In the executive pathway, the high efficiency of top-down processing and execution is guaranteed, which may lay a certain foundation for the improvement in distraction suppression ability.

### Changes in the regional congruence state of local areas and distraction suppression ability

ReHo reflects the synchronicity of activity between the time series of a given voxel and its neighbors and is evidence of the relative independence of various parts of the brain. After anodal tDCS, the ReHo in the left precuneus remains significantly higher. The changes of ReHo in the left precuneus between the two sessions significantly negatively correlated with the change in the distractor effect. The results suggest that the greater ReHo increasement is relative to the more effective distraction suppression. Anatomical studies have found that the precuneus plays a pivotal role in various integrated functions, which are not simply an extension of the visual spatial processing maintained by the parietal cortex (Fransson and Marrelec, 2008). In attention, the precuneus is also be proved to related with the attention network. Evidence from stroke patients suggested that the decreased ReHo in the precuneus is associated with attention processing impairments, whereas increased ReHo in precuneus indicated relatively good behavioral performance (Peng et al., 2016). Despite all of the participants in our study are normal human, Peng’ s study could still provide reasonable interpretation for our results, i.e., increasement ReHo in the precuneus might lay the foundation to the effective distraction suppression, which also demonstrated the function of the precuneus for visual integration processing (Cavanna and Trimble, 2006; Fransson and Marrelec, 2008). The change in ReHo in the right frontal region and rPG positively correlated with the change in the distractor effect. The rPG lies at the edge of the frontal cortex, which facilitates senior processing, and deals with primary sensory perception. Earlier studies suggested that the rPG could merge the spatial coding of the oculomotor and sensorimotor muscles and showed that an increase in cerebral blood flow correlated with increases in reaction time induced by inconsistent brain responses (Iacoboni et al., 1997). The right frontal region also lies in BA 4 and belongs to the SMN, which may contribute to controlling voluntary movements. Together, these two areas may also be involved in distraction suppression processing, although the ReHo changed non-significantly.

### Limitations

In this study, the reference electrode was placed on the right cheek to avoid any confounding effect from other brain regions. However, the position of the electrode may make difference to the effect induced by tDCS. Therefore, the results of this study are of limited explanatory value for those studies of arranging the reference electrode over the contralateral orbital or the symmetrical region. Second, we using the offline-tDCS design which may affect the observed tDCS effect due to the possible attenuation of tDCS intervention, if technically possible, concurrent tDCS-fMRI design will be conducted in subsequent studies. Meanwhile, we didn’t evaluate the visual acuity before the visual search task to ensure the perfect task completion, fortunately all participants made their best effort to complete the visual section (as the ACC of each session surpassed 0.95). For rigor, visual acuity should be assessed first in subsequent visual-task related studies. In addition, we chose to use single-blind design in this study, which may show a lack of rigor to some extent. The major problem in single-blind design is the potential different handlings for the data derive from the anodal-tDCS stage and the sham stage, which may due to the expectation for the anodal-tDCS’ effect. However, single-blind design cannot affect the objective results on condition that the standardized analysis procedure performed. In this study, data from both anodal-tDCS and sham stage received entirely consistent analysis, therefore the single-blind design can fully satisfy the needs of verifying experimental hypotheses. Finally, the current study only focused on the changes of local brain states, and did not explore the large-scale network alteration when behavioral performance was enhanced by tDCS, which will be further improved in subsequent studies.

### Summary

In conclusion, our results suggest that anodal tDCS performed on the lDLPFC can alter the local states of several brain regions and effectively improve the ability to inhibit distractions. The correlation analysis revealed that a certain correlation exists between the change in distraction suppression ability and the alterations of the local states of different brain areas. After anodal tDCS, the decreased spontaneous activity in the left parietal region indicates that the brain had been in a state of involuntary movement inhibition. Meanwhile, the increased ReHo in the left precuneus might make a precondition for improvement of attention and visual processing, thus led an improved performance of distraction suppression. The correlation results suggest that the alteration of the local brain state probably provides an opportunity for the improvement of the ability to overcome irrelevant information in a visual search task. The results demonstrated a state of the brain with inclination to controlling, execution and preparation when the distractor was suppressed more effectively, which may provide a new insight into the interpretation of how the local state of the brain changes during behavioral performance enhancement.

## Conclusion

Anodal tDCS on the lDLPFC can change the local activity state of several brain regions and improve distraction suppression ability. This behavioral improvement is closely correlated with the local state changes in multiple areas, which provide a direct and efficient approach to enhance the performance for overcoming irrelevant information.

## Data availability statement

The raw data supporting the conclusions of this article will be made available by the authors, without undue reservation.

## Ethics statement

The studies involving human participants were reviewed and approved by the Ethics Committee of the Liaoning Normal University Research Center of Brain and Cognitive Neuroscience. Written informed consent for participation was not required for this study in accordance with the national legislation and the institutional requirements.

## Author contributions

DZ: conceptualization, investigation, data curation, methodology, formal analysis, software, validation, visualization, and writing – original draft. JL and LF: data curation. QL: conceptualization, investigation, project administration, resources, supervision, and writing – review and editing. All authors contributed to the article and approved the submitted version.

## Footnotes

1
http://www.rfmri.org/dpabi
